# Identifying Patients with Bacteremia in Community-Hospital Emergency Rooms: A Retrospective Cohort Study

**DOI:** 10.1371/journal.pone.0148078

**Published:** 2016-03-29

**Authors:** Taro Takeshima, Yosuke Yamamoto, Yoshinori Noguchi, Nobuyuki Maki, Koichiro Gibo, Yukio Tsugihashi, Asako Doi, Shingo Fukuma, Shin Yamazaki, Eiji Kajii, Shunichi Fukuhara

**Affiliations:** 1 Department of Healthcare Epidemiology, School of Public Health in the Graduate School of Medicine, Kyoto University, Kyoto, Japan; 2 Division of Community and Family Medicine, Center for Community Medicine, Jichi Medical University, Tochigi, Japan; 3 Institute for Advancement of Clinical and Translational Science, Kyoto University Hospital, Kyoto, Japan; 4 Department of General Internal Medicine, Japanese Red Cross Nagoya Daini Hospital, Aichi, Japan; 5 Department of Emergency Medicine, Shizuoka General Hospital, Shizuoka, Japan; 6 Biostatistics Center, Kurume University, Kurume, Fukuoka, Japan; 7 Department of Home Care Medicine, Tenri Hospital, Nara, Japan, Tenri Hospital, Nara, Japan; 8 Department of General Internal Medicine and Infectious Diseases, Kobe City Medical Center General Hospital, Hyogo, Japan; 9 Center for Environmental Health Sciences, National Institute for Environmental Studies, Ibaraki, Japan; 10 Center for Innovative Research for Communities and Clinical Excellence (CIRC2LE), Fukushima Medical University, Fukushima, Japan; University of Parma, ITALY

## Abstract

**Objectives:**

(1) To develop a clinical prediction rule to identify patients with bacteremia, using only information that is readily available in the emergency room (ER) of community hospitals, and (2) to test the validity of that rule with a separate, independent set of data.

**Design:**

Multicenter retrospective cohort study.

**Setting:**

To derive the clinical prediction rule we used data from 3 community hospitals in Japan (derivation). We tested the rule using data from one other community hospital (validation), which was not among the three “derivation” hospitals.

**Participants:**

Adults (age ≥ 16 years old) who had undergone blood-culture testing while in the ER between April 2011 and March 2012. For the derivation data, n = 1515 (randomly sampled from 7026 patients), and for the validation data n = 467 (from 823 patients).

**Analysis:**

We analyzed 28 candidate predictors of bacteremia, including demographic data, signs and symptoms, comorbid conditions, and basic laboratory data. Chi-square tests and multiple logistic regression were used to derive an integer risk score (the “ID-BactER” score). Sensitivity, specificity, likelihood ratios, and the area under the receiver operating characteristic curve (i.e., the AUC) were computed.

**Results:**

There were 241 cases of bacteremia in the derivation data. Eleven candidate predictors were used in the ID-BactER score: age, chills, vomiting, mental status, temperature, systolic blood pressure, abdominal sign, white blood-cell count, platelets, blood urea nitrogen, and C-reactive protein. The AUCs was 0.80 (derivation) and 0.74 (validation). For ID-BactER scores ≥ 2, the sensitivities for derivation and validation data were 98% and 97%, and specificities were 20% and 14%, respectively.

**Conclusions:**

The ID-BactER score can be computed from information that is readily available in the ERs of community hospitals. Future studies should focus on developing a score with a higher specificity while maintaining the desired sensitivity.

## Introduction

Delaying treatment of bacteremia can be fatal. [1] Thus, clinicians need to very quickly identify patients who have bacteremia, a diagnosis that is confirmed with blood-culture results. [2]

However, clinicians may suspect that sepsis is present in too many patients in whom it is, in fact, absent. If blood cultures are used for all patients in whom bacteremia might be suspected, then most of the results will be negative. Specifically, in previous studies 4–7% of blood-culture results have been positive. [3–4] Blood-culture analyses can be costly, resulting in a 20% increase in total hospital costs for patients with false-positive blood-culture results. [5–7] Moreover, the results of blood-culture testing may not affect clinical decisions. [8–9] Unnecessary blood cultures of course waste medical resources and healthcare workers’ time, and expose them and their patients to unnecessary risks.

Furthermore, because of the time required, blood-culture results do not inform the decision of whether or not to start treatment. That decision is challenging also because the clinical presentation of bacteremia varies greatly, depending on the cause of the infection. [10] Physicians often overestimate a patient's likelihood of having bacteremia. In the emergency room (ER) of a community hospital, if one could identify those patients who are very likely to have community acquired bacteremia, then it might be possible to avoid, at least in a few cases, unnecessary punctures for blood samples, unnecessary antibiotic therapy, and unnecessary admission to hospital. [11]

Attempts to devise a quick procedure to identify patients with bacteremia have had only limited success. [12] Some are useful only in elderly patients [13] or only in those with urinary-tract infections [14] or with pneumonia. [15] Others require complex calculations, [16] or use rare or difficult-to-obtain measurements. [17, 18] While some studies reported rules for predicting hospital-acquired bacteremia, [3, 13, 16, 19–21] at least three procedures have been developed to identify community acquired bacteremia among ER patients. [17, 18, 22] They were developed using data from one university hospital each, so their utility in ERs of various community hospitals is unclear. In addition, they use bands or procalcitonin values, which are usually not available in ERs of community hospitals, [17, 18] and their development did not include validation studies. [18, 22]

The objective of this study was to develop a new prediction rule to identify bacteremia, which will overcome some of the limitations of prior studies. Here we report on the development and testing of a highly sensitive procedure that uses only information that is readily available in ERs of community hospitals, to identify patients who have community acquired bacteremia.

## Methods

### Study design

In this multicenter retrospective cohort study we derived and evaluated a clinical prediction rule. First we used clinical data from 3 hospitals (the “derivation” data) to develop a procedure for identifying patients in the ER who have bacteremia, and then we used data from one other hospital (the “validation” data) to test that procedure.

### Setting

The setting was 4 community hospitals in Japan, all of which receive patients on an emergency basis and all of which provide primary, secondary, and tertiary care. The Japanese Red Cross Nagoya Daini Hospital and Tenri Hospital have transplant wards, and Okinawa General Hospital has a Level I trauma center. The data were obtained from patients aged 16 years and older who had undergone blood-culture testing while in the ER between 1 April 2011 and 31 March 2012. In these ERs, physicians drew blood for cultures from different sites each time, and with no time interval between blood samples. In 2009 in Japan, the median percentage of blood-culture tests involving multiple sets of samples was 67.2%. [23] In 2014, it became more common to take multiple sets of samples for blood cultures, because their cost began to be covered under the national health insurance system. [24] In this study, we excluded patients from whom there was only one sample for blood culture.

Derivation data came from the Japanese Red Cross Nagoya Daini Hospital, Okinawa General Hospital, and Shizuoka General Hospital. Those hospitals have, respectively, 812 beds, 550 beds, and 720 beds. The numbers of ER patients who underwent blood-culture tests were, 2264 (4.9% of 45,779 visits in one year), 4180 (11.3% of 37,106 visits in one year), and 582 (6.6% of 9738 visits in one year) for those three hospitals, respectively.

**Fig 1:**
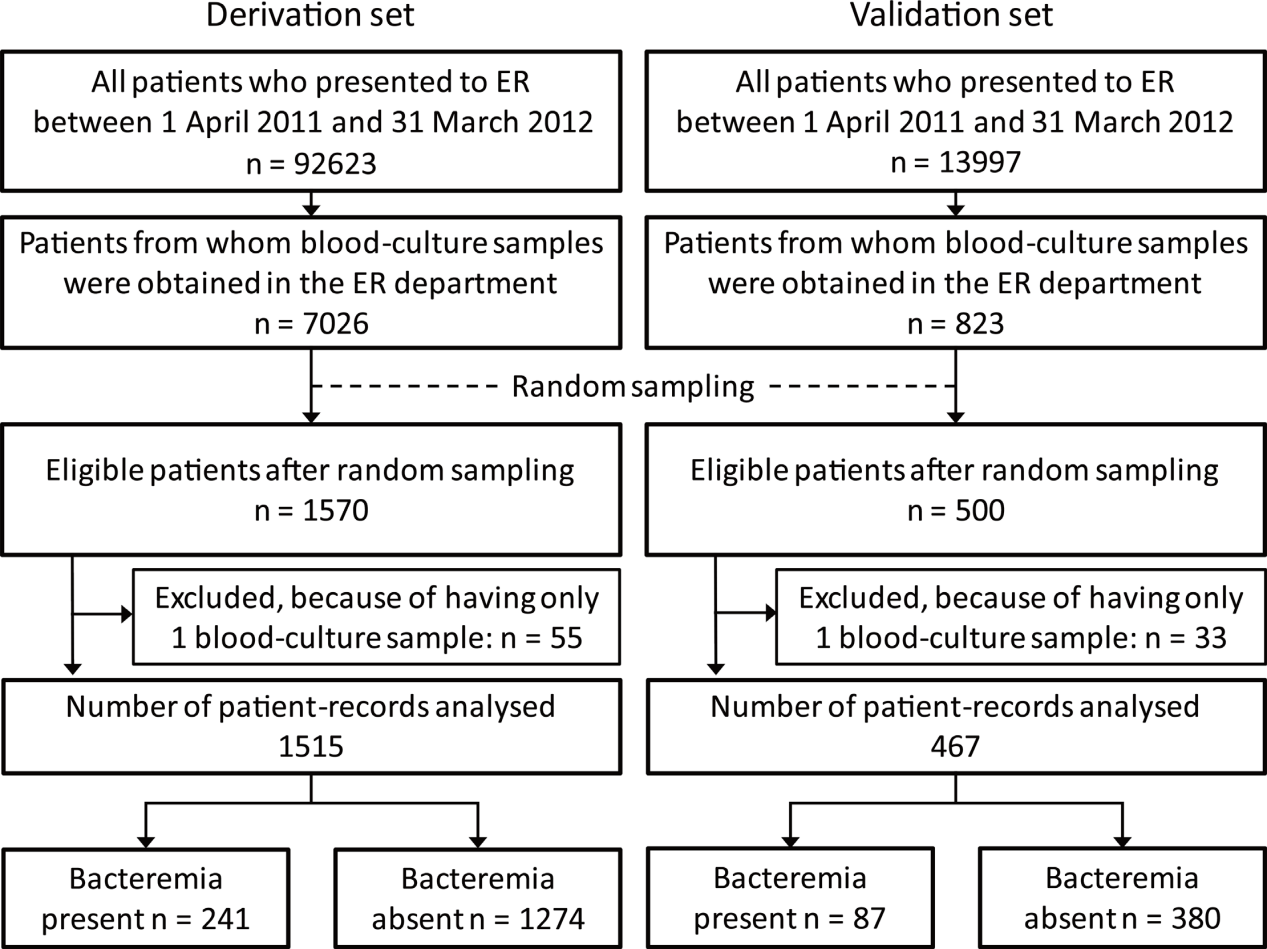
Flow diagram of participants. Fig 1 shows the flow diagram of participants in this study. We began with records of all patients who presented to the ER between April 2011 and March 2012. After the random sampling and exclusions indicated in the Figure, we analysed data from 1515 patients for the derivation set, and data from 467 patients for the validation set.

One very common practice is to include at least 10 cases with the outcome (i.e., bacteremia) for each potential predictor in a multivariable model. We considered that we might need to use up to 15 predictors, and so, with “10 cases per predictor” in mind, we expected to need about 150 cases of bacteremia. Next, to be conservative, we presumed that only about 10% of the patients to be studied in fact had bacteremia. Thus, we estimated that we would need to study records from about 1500 patients (as 10% of 1500 is 150). Sampling approximately equally from the three hospitals would result in about 500 records from each hospital. We assumed that some of the records would be unusable (because of excessive missing data, inconsistencies, etc.) and so we sampled, randomly, somewhat more than 500 from two of the hospitals (in Nagoya and in Okinawa), and we used as many of those sampled records as possible. Regarding the third hospital (Shizuoka General Hospital), the total number available was not far from 500 (it was 582), so we used data from all 582. Thus, we collected information from a total of 1570 patients: 505 sampled randomly from those in Nagoya, 483 sampled randomly from those in Okinawa, and all 582 from Shizuoka. Of those 1570 patients, 55 underwent only one blood-culture test. We excluded those 55 and analysed the data from the remaining 1515 (96.5% of 1570) (Fig 1).

Validation data came from Tenri Hospital (in Nara Prefecture), which has 815 beds. The number of ER patients who underwent blood-culture tests was 823 (5.9% of 13,997 visits in one year). We randomly chose 500 patients, then excluded 33 because they had undergone only one test, and analysed data from the remaining 467 (93.4% of 500) (Fig 1).

### Candidate predictors

In choosing candidate predictors, we excluded those that could not be readily obtained while a patient was in the ER of a community hospital. We also took into account the results of recent studies [3, 12–22, 25, 26] and had discussions with 4 physicians, each of whom had more than 10 years of experience in clinical practice. The candidate predictors (Table 1) included demographic data, signs and symptoms, mental status, comorbid conditions, and laboratory data (measured within 12 hours of arrival at the ER). Demographic data were gender, age (≥ 65 years), fever, chills including shaking chills, vomiting (which did not include only nausea), dyspnea (including orthopnea), and spontaneous abdominal pain. Whether patients had fever, chills, vomiting, dyspnea, or abdominal pain at the initial visit was evaluated by the doctors who worked in the ER of each hospital. Comorbid conditions were hemodialysis, stroke (including cerebral hemorrhage, cerebral infarction, subarachnoid hemorrhage, and a history of those conditions), diabetes, cirrhosis (including any stage of cirrhosis), malignancy (including any stage of malignancy and past history of malignancy), urinary catheter, and the presence of an internal device (i.e, an implanted central-venous access device, an implanted pacemaker, an implanted cardioverter defibrillator, or an artificial valve). Comorbid conditions were assessed by information in the medical records and by medical interviews done by the doctors at the initial visit. Vital signs were altered mental status, which was defined as a score ≤ 14 on the Glasgow coma scale or a score ≥ 1 on the Japan coma scale, body temperature ≥ 38.0 degrees C, systolic blood pressure < 90 mmHg, pulse rate ≥ 100/min, respiratory rate ≥ 20/min, heart murmur, and focal abdominal sign including tenderness, rebound tenderness, and muscular defense (guarding). Laboratory data were white blood cell count ≥ 15,000/μL, hemoglobin < 10 mg/dL, platelets < 150,000/μL, blood urea nitrogen (BUN) ≥ 20 mg/dL, creatinine (Cre) ≥ 1.5 mg/dL, lactate dehydrogenase (LDH) ≥ 400 IU/L, and C-reactive protein (CRP) ≥ 10 mg/dL. From each participant’s medical record we collected data regarding those candidate predictors.

**Table 1:** Demographic and clinical characteristics.

	Derivation			Validation			
	(n = 1515)			(n = 467)			
Variable	n		(%)	n		(%)	p
**Demographics, etc.**							
Gender, male	803		(53.0)	255		(54.6)	0.54
Age, mean ±SD	70.1	±	18.9	71.2	±	16.9	0.27^†^
Arrival by ambulance	742		(49.0)	268		(57.4)	<0.01
Final diagnosis							
Pneumonia	432		(28.5)	143		(30.6)	0.38
Urinary tract infection	225		(14.9)	65		(13.9)	0.62
Cellulitis	90		(5.9)	21		(4.5)	0.24
Biliary tract infection	73		(4.8)	33		(7.1)	0.06
Endocarditis	14		(0.9)	1		(0.2)	0.12^‡^
Others	817		(53.9)	258		(55.2)	0.62
**Bacteremia**	241		(15.9)	87		(18.6)	0.17
**Candidate predictors**							
**History**							
Age ≥ 65 years old	1040		(68.6)	347		(74.3)	0.02
Male	803		(53.0)	255		(54.6)	0.54
Fever	893		(59.0)	351		(75.6)	<0.01
Chills	206		(13.6)	148		(32.7)	<0.01
Vomiting	150		(9.9)	64		(14.2)	0.01
Dyspnea	277		(18.3)	89		(19.6)	0.54
Abdominal pain	173		(11.4)	72		(15.9)	0.01
**Comorbidities, etc.**							
Hemodialysis	41		(2.7)	8		(1.7)	0.22
Stroke	216		(14.4)	58		(12.4)	0.29
Diabetes	251		(16.7)	103		(22.1)	<0.01
Cirrhosis	46		(3.1)	10		(2.2)	0.31
Malignancy	284		(18.9)	107		(23.0)	0.05
Urinary catheter	24		(1.6)	8		(1.7)	0.85
Device	58		(3.9)	31		(6.7)	<0.01
**Signs**							
Altered mental status	387		(26.3)	116		(25.2)	0.64
Body temperature ≥ 38°C	685		(46.3)	219		(48.6)	0.39
Systolic blood pressure < 90 mmHg	116		(7.9)	22		(5.0)	0.04
Pulse rate ≥ 100/min	652		(44.5)	200		(46.0)	0.58
Respiratory rate ≥ 20/min	829		(72.5)	169		(64.5)	<0.01
Heart murmur	111		(7.5)	25		(5.5)	
Focal abdominal sign	185		(12.5)	76		(16.8)	0.02
**Laboratory data**							
White blood cells ≥ 15,000/μL	287		(19.0)	68		(15.0)	0.05
Hemoglobin < 10 mg/dL	277		(18.4)	93		(20.0)	0.43
Platelets < 150,000/μL	427		(28.3)	123		(26.4)	0.42
Blood urea nitrogen (BUN) ≥ 20 mg/dL	681		(45.2)	190		(40.7)	0.08
Creatinine ≥ 1.5 mg/dL	249		(16.5)	83		(17.8)	0.54
Lactate dehydrogenase (LDH) ≥ 400 IU/L	155		(13.4)	38		(8.2)	<0.01
C-reactive protein (CRP) ≥ 10 mg/dL	453		(31.2)	158		(34.0)	0.27

SD: standard deviation

*p*-values were calculated using chi-square test

† *t-*test, and

‡ Fisher's exact test

### Definition of true bacteremia

True bacteremia was defined as growth of known pathogenic bacteria in 1 blood culture or as growth of common skin pathogens (i.e., coagulase-negative Staphylococcus species, diphtheroids, Bacillus species, Propionibacterium species, or micrococci) in 2 blood cultures. [19] True bacteremia was distinguished from contamination by the judgment of 2 physicians working independently, each of whom had more than 10 years of clinical experience. They referred to the results of at least 2 blood cultures and to the patient’s clinical course. [27] We calculated kappa to quantify the agreement between those 2 physicians. When the 2 physicians’ first judgements did not agree, they discussed the case until they reached agreement.

### Data collection

For data collection we used a standardized clinical research form. For the derivation data, records were in electronic form at three institutions. The data were collected by four physicians. For the validation data, the one institution from which data were collected had records on paper only, and the data in those records were collected by 18 physicians. Records with missing values and outliers were checked by the first author (TT).

### Development of clinical prediction rule

First we used the chi-square test to identify variables associated with true bacteremia. Those variables for which the result of the chi-square test was statistically significant (p < 0.05) were then entered into a multiple logistic regression model. Only predictors with a p value of less than 0.05 were kept in the final model. We then used regression coefficients to make the score-based prediction rule, as previously discussed by Moons et al. [28] For each variable, we divided its beta coefficient by the beta coefficient for temperature, converted the result to an integer, and then used that for the score. Then we divided the patients into five groups by risk-score, and we compared the observed percentages of bacteremia among those groups. The overall discriminative power of the model was quantified as the area under the receiver-operating characteristic (ROC) curve. [29] Calibration, that is, the agreement between the predicted outcomes and the observed outcomes, was evaluated by using the Hosmer–Lemeshow chi-square statistic [30] and the slope and intercept of the calibration plot. [31] In the calibration plot, the predictions are on the x-axis and the observations are on the y-axis. An ideal calibration line has a slope of 1 and an intercept of 0. [31]

### Assessment of test performance

For all possible cut-off scores we computed the sensitivity, the specificity, and the likelihood ratios for positive and negative test results. [29]

### Validation testing

For internal validation testing we used the bootstrap method (1000 iterations) with the derivation data. [32] For each bootstrap sample, patients were drawn randomly (with replacement) from the derivation data set, and each bootstrap sample was the same size as the derivation sample. For each iteration the model was refitted, and discrimination was again quantified as the area under the ROC curve (AUC).

For external validation testing we used the validation data. We computed the total risk score of each patient in the validation data set. Then, for each of the categories described above we computed the percentages of patients who had bacteremia. We also computed the AUC, the sensitivity and specificity, and the likelihood ratios, also as described above. Further, we compared AUCs between the model developed as described above and a model incorporating only temperature.

### Software, and research ethics

The data were analyzed with Stata version 11.2 (Stata Corp., College Station, Texas). The Research Ethics Committee of Kyoto University approved this study (assessment number E1382). The data were anonymized before analyses.

## Results

### Patient characteristics

Table 1 shows demographic and clinical characteristics of the patients from whom the derivation and validation data were obtained. The differences between those two groups of patients were small. The two groups significantly differed with regard to 12 characteristics: age ≥ 65, arrival by ambulance, fever, chills, vomiting, abdominal pain, diabetes, devices, systolic blood pressure < 90 mm Hg, respiratory rate ≥ 20/min, focal abdominal sign, and LDH ≥ 400 IU/L.

### Bacteremia

The derivation data included 241 cases of bacteremia: 70 in Nagoya, 42 in Okinawa, and 129 in Shizuoka. The validation data included 87 cases of bacteremia. In the derivation data, 419 pathogens were isolated by blood culture, of which 274 were judged to be true pathogens, with the others judged to be contamination. In the validation data, 121 pathogens were also isolated, of which 91 were judged to be true pathogens. The Kappa statistic for the two reviewers’ judgments was 0.94 (95%CI, 0.92–0.96).

### Clinical prediction rule

With the exceptions of data on respiratory rate and lactate dehydrogenase (LDH), very few data were missing. As shown in Table 2, 19 of the 28 candidate predictors had statistically significant associations with bacteremia: age ≥ 65 years, 9 signs and symptoms, 3 comorbid conditions, and 6 blood-test findings. The results of multivariate analysis (with n = 1288) indicated that 11 predictors were associated with bacteremia: age ≥ 65 years old, chills, vomiting, altered mental status, temperature ≥ 38°C, systolic blood pressure < 90 mm Hg, focal abdominal sign, white blood cell count ≥ 15,000/μL, platelets < 150,000/μL, BUN ≥ 20 mg/dL, and CRP ≥ 10 mg/dL. Those predictors and their integer scores are shown in Table 3. The sum of those scores can range from 0 to 12. Because that sum is to be used to ***id***entify ***bacter***emia in ERs, we call it the ID-BactER score. For the patients in the derivation set, the mean ID-BactER score was 3.3 (95% CI, 3.2–3.4, median: 3, interquartile range: 2–4, range of observed scores: 0–9).

**Table 2:** Univariate associations between candidate predictors and true bacteremia in the derivation set,

	missing	Bacteremia			Non-bacteremia					
		(n = 241)			(n = 1274)					
Candidate predictors	%	exposed	total	(%)	exposed	total	(%)	OR	95% CI	p
**History**										
Age ≥ 65 years old	0.0	183	241	(75.9)	857	1274	(67.3)	1.54	1.12–2.11	<0.01
Male	0.0	129	241	(53.6)	674	1274	(52.9)	1.03	0.78–1.35	0.86
Fever	0.1	164	241	(68.0)	729	1273	(57.3)	1.59	1.19–2.13	< 0.01
Chills	0.1	78	241	(32.4)	128	1273	(10.1)	4.28	3.09–5.93	< 0.01
Vomiting	0.0	42	241	(17.4)	108	1274	(8.5)	2.28	1.55–3.35	< 0.01
Dyspnea	0.1	36	241	(14.9)	241	1273	(18.9)	0.75	0.51–1.10	0.14
Abdominal pain	0.1	38	241	(15.8)	135	1273	(10.6)	1.58	1.07–2.33	0.02
**Comorbidities, etc.**									
Hemodialysis	0.7	6	241	(2.5)	35	1263	(2.8)	0.90	0.38–2.10	0.81
Stroke	0.7	32	241	(13.4)	184	1263	(14.6)	0.90	0.60–1.34	0.60
Diabetes	0.7	49	241	(20.4)	202	1264	(16.0)	1.34	0.95–1.90	0.10
Cirrhosis	0.7	8	241	(3.3)	38	1263	(3.0)	1.11	0.52–2.36	0.80
Malignancy	0.7	62	241	(25.7)	222	1264	(17.6)	1.63	1.18–2.24	< 0.01
Urinary catheter	0.8	9	241	(3.7)	15	1262	(1.2)	3.23	1.43–7.30	< 0.01
Devices	0.7	16	241	(6.6)	42	1263	(3.3)	2.07	1.15–3.71	0.01
**Signs**									
Altered mental status	2.9	83	232	(35.8)	304	1239	(24.5)	1.71	1.27–2.31	< 0.01
Body temperature ≥ 38°C	2.2	156	238	(65.5)	529	1243	(42.6)	2.57	1.92–3.43	< 0.01
Systolic blood pressure < 90 mmHg	2.8	38	231	(16.5)	78	1242	(6.3)	2.94	1.94–4.45	< 0.01
Pulse rate ≥ 100/min	3.2	121	228	(53.1)	531	1238	(42.9)	1.51	1.13–2.00	< 0.01
Respiratory rate ≥ 20/min	24.6	121	160	(75.6)	708	983	(72.0)	1.21	0.82–1.77	0.34
Heart murmur	2.2	17	237	(7.2)	94	1244	(7.6)	0.95	0.56–1.61	0.84
Focal abdominal sign	2.2	52	236	(22.0)	133	1245	(10.7)	2.36	1.66–3.37	< 0.01
**Laboratory data**									
White blood cells ≥ 15,000/μL	0.5	67	241	(27.8)	220	1267	(17.4)	1.83	1.34–2.51	< 0.01
Hemoglobin < 10 mg/dL	0.5	59	241	(24.5)	218	1267	(17.2)	1.56	1.12–2.16	< 0.01
Platelets < 150,000/μL	0.5	107	241	(44.4)	320	1267	(25.3)	2.36	1.78–3.14	< 0.01
BUN ≥ 20 mg/dL	0.6	148	241	(61.4)	533	1265	(42.1)	2.19	1.65–2.90	< 0.01
Cre ≥ 1.5 mg/dL	0.7	62	241	(25.7)	187	1264	(14.8)	1.99	1.44–2.77	< 0.01
LDH ≥ 400 IU/L	23.7	34	215	(15.8)	121	941	(12.9)	1.27	0.84–1.92	0.25
CRP ≥ 10 mg/dL	4.2	121	238	(50.8)	332	1213	(27.4)	2.74	2.07–3.64	< 0.01

OR = odds ratio, 95%CI = 95% confidence interval, BUN = blood urea nitrogen, Cre = creatinine, LDH = lactate dehydrogenase, CRP = C-reactive protein

**Table 3:** Multivariate analysis (n = 1288) and scoring.

	OR	95% CI	p	β	score
**History**					
Age ≥ 65 years old	1.64	(1.07–2.52)	0.02	0.49	1
Chills	4.61	(3.03–7.01)	< 0.01	1.53	2
Vomiting	1.68	(1.02–2.76)	0.04	0.52	1
**Signs**					
Altered mental status	1.62	(1.10–2.37)	0.01	0.48	1
Body temperature ≥ 38°C	2.34	(1.56–3.50)	< 0.01	0.85	1
Systolic blood pressure < 90 mmHg	2.28	(1.34–3.90)	< 0.01	0.83	1
Focal abdominal sign	2.64	(1.51–4.61)	< 0.01	0.97	1
**Laboratory data**						
White blood cells ≥ 15,000/μL	2.06	(1.38–3.08)	< 0.01	0.72	1
Platelets < 150,000/μL	1.88	(1.31–2.68)	< 0.01	0.63	1
BUN ≥ 20 mg/dL	1.68	(1.12–2.50)	0.01	0.52	1
CRP ≥ 10 mg/dL	2.77	(1.94–3.95)	< 0.01	1.02	1

OR = odds ratio, 95%CI = 95% confidence interval, BUN = blood urea nitrogen, Cre = creatinine, LDH = lactate dehydrogenase, CRP = C-reactive protein

**Fig 2:**
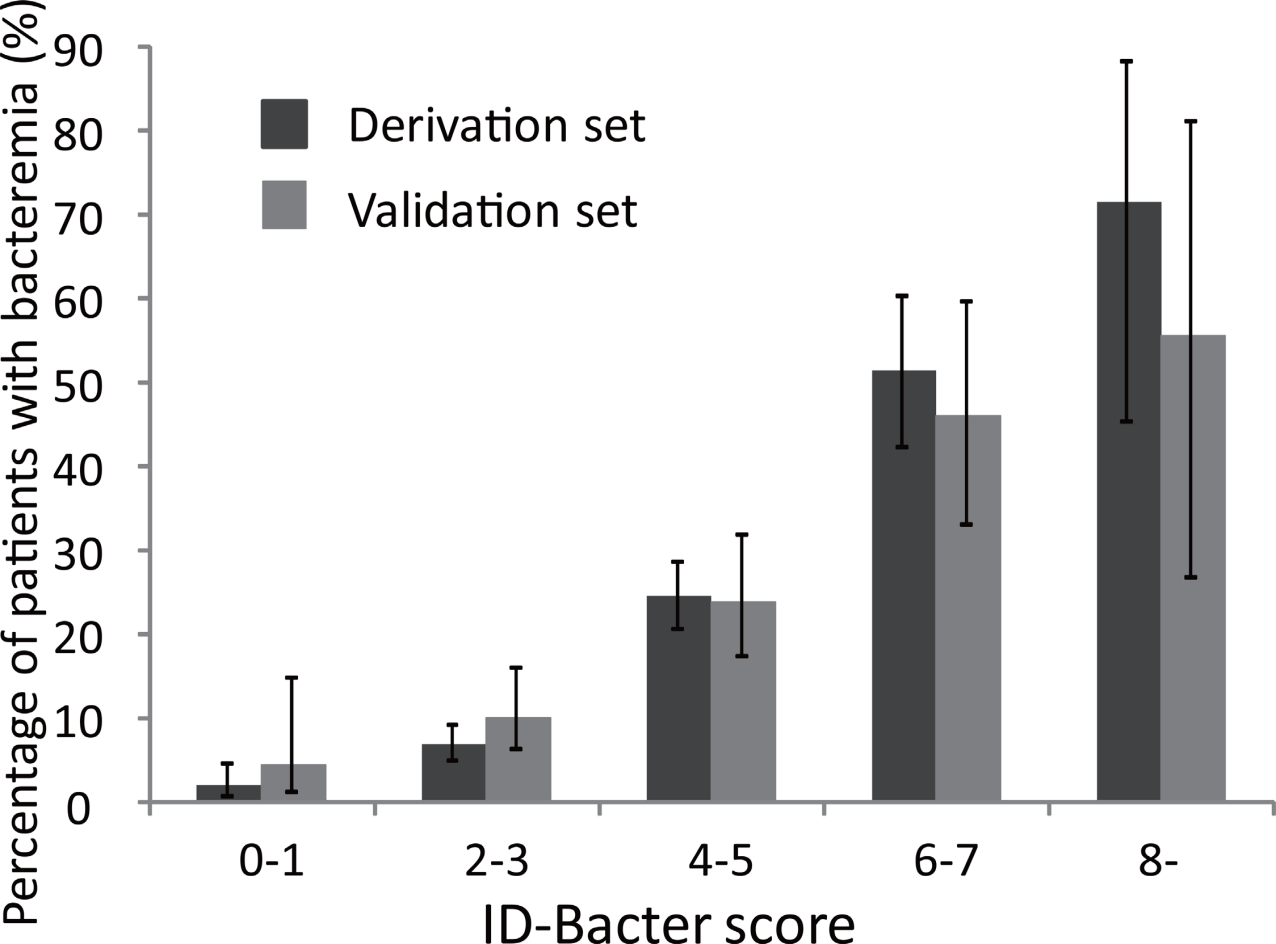
Percentages of patients with true bacteremia, by ID-BactER score. Fig 2 shows, for each of five categories defined by ID-BactER score, the percentage of patients in that category who had true bacteremia.

**Fig 3:**
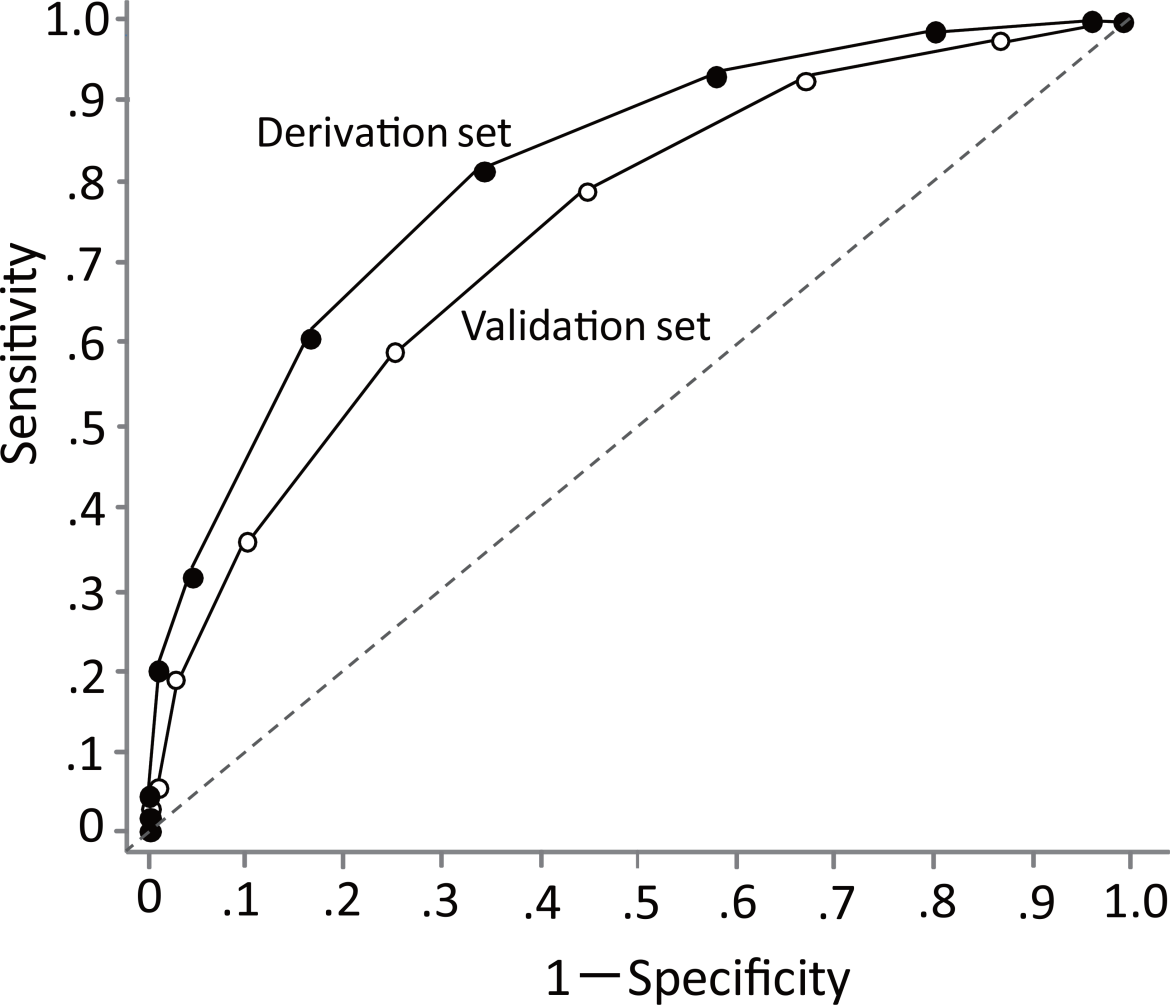
Fig 3 shows the ROC curves for the ID-BactER score. **ROC curves of ID-BactER score.** The ●s indicate results from the derivation set, and the ○s indicate results from the validation set. The areas under the curves are 0.80 (95% CI, 0.77–0.83) for the derivation set and 0.74 (0.68–0.80) for the validation set.

Each patient in the derivation set was assigned to one of five categories, by ID-BactER score. The lower ID-BactER score categories had lower percentages of patients with bacteremia. Categories defined by higher ID-BactER scores had higher percentages of patients with bacteremia (Fig 2). Specifically, bacteremia was detected in 1.8% (4/222) of patients with a score of 0 or 1; in 6.7% (36/537) with a score of 2 or 3; in 24.2% (105/434) with a score of 4 or 5; in 51.3% (59/115) with a score of 6 or 7; and in 71.4% (10/14) with a score greater than 7. The AUC was 0.80 (95% CI, 0.77–0.83, Fig 3). The Hosmer-Lemeshow chi-square statistic was 5.84 (p = 0.66). The calibration slope was 1.02 and the intercept was -0.01. Sensitivities, specificities, and likelihood ratios for possible cut-off scores are shown in the upper part of Table 4. With 2 as the cutoff score, the sensitivity was 98%, the specificity was 20%, the positive likelihood ratio was 1.22, the negative likelihood ratio was 0.10, and the percentage of false negatives was 1.8% (4/222).

**Table 4:** Performance of the ID-BactER score.

**Derivation set**												
cut-off	Se	95% CI	Sp	95% CI	LR+	95% CI	LR-	95% CI	PPV	95% CI	NPV	95% CI
≥9	0.01	(0.00–0.04)	1.00	(1.00–1.00)	.	(.—.)	0.99	(0.98–1.00)	1.00	(0.16–1.00)	0.84	(0.82–0.86)
≥8	0.05	(0.02–0.09)	1.00	(0.99–1.00)	12.94	(4.10–40.89)	0.96	(0.93–0.99)	0.71	(0.42–0.92)	0.84	(0.82–0.86)
≥7	0.20	(0.15–0.26)	0.98	(0.98–0.99)	13.10	(7.62–22.52)	0.81	(0.76–0.87)	0.72	(0.59–0.83)	0.86	(0.84–0.88)
≥6	0.32	(0.26–0.39)	0.95	(0.93–0.96)	5.95	(4.35–8.15)	0.72	(0.65–0.79)	0.53	(0.45–0.62)	0.88	(0.86–0.90)
≥5	0.60	(0.53–0.67)	0.83	(0.81–0.85)	3.53	(2.98–4.19)	0.48	(0.41–0.57)	0.41	(0.35–0.46)	0.92	(0.90–0.93)
≥4	0.81	(0.75–0.86)	0.65	(0.62–0.68)	2.32	(2.09–2.57)	0.29	(0.22–0.38)	0.31	(0.27–0.35)	0.95	(0.93–0.96)
≥3	0.93	(0.88–0.96)	0.42	(0.39–0.45)	1.59	(1.49–1.69)	0.18	(0.11–0.29)	0.23	(0.21–0.26)	0.97	(0.95–0.98)
≥2	0.98	(0.95–0.99)	0.20	(0.17–0.22)	1.22	(1.18–1.26)	0.10	(0.04–0.25)	0.19	(0.17–0.22)	0.98	(0.95–1.00)
≥1	1.00	(0.98–1.00)	0.04	(0.03–0.05)	1.04	(1.02–1.05)	0.00	(.—.)	0.17	(0.15–0.19)	1.00	(0.91–1.00)
**Validation set**															
cut-off	Se	95% CI	Sp	95% CI	LR+	95% CI	LR-	95% CI	PPV	95% CI	NPV	95% CI
≥9	0.03	(0.00–0.10)	1.00	(0.98–1.00)	.	(.—.)	0.97	(0.94–1.01)	1.00	(0.16–1.00)	0.81	(0.76–0.84)
≥8	0.06	(0.02–0.15)	0.99	(0.97–1.00)	5.03	(1.38–18.30)	0.95	(0.89–1.01)	0.56	(0.21–0.86)	0.81	(0.77–0.85)
≥7	0.19	(0.12–0.30)	0.97	(0.94–0.99)	6.71	(3.05–14.75)	0.83	(0.74–0.93)	0.63	(0.41–0.81)	0.83	(0.79–0.87)
≥6	0.36	(0.26–0.48)	0.90	(0.86–0.93)	3.64	(2.33–5.68)	0.71	(0.59–0.84)	0.47	(0.34–0.61)	0.85	(0.81–0.89)
≥5	0.58	(0.47–0.69)	0.75	(0.70–0.80)	2.38	(1.82–3.13)	0.55	(0.42–0.72)	0.37	(0.29–0.46)	0.88	(0.83–0.92)
≥4	0.78	(0.67–0.86)	0.57	(0.51–0.63)	1.82	(1.52–2.16)	0.39	(0.25–0.59)	0.31	(0.25–0.38)	0.91	(0.86–0.95)
≥3	0.92	(0.83–0.97)	0.32	(0.27–0.37)	1.35	(1.23–1.50)	0.24	(0.11–0.54)	0.25	(0.20–0.31)	0.94	(0.88–0.98)
≥2	0.97	(0.90–1.00)	0.14	(0.10–0.18)	1.13	(1.07–1.20)	0.19	(0.05–0.76)	0.22	(0.18–0.27)	0.96	(0.85–0.99)
≥1	1.00	(0.94–1.00)	0.04	(0.02–0.07)	1.04	(1.02–1.07)	0.00	(.—.)	0.21	(0.17–0.25)	1.00	(0.75–1.00)

Se = sensitivity, Sp = specificity, 95% CI = 95% confidence interval, LR+ = positive likelihood ratio, LR- = negative likelihood ratio, PPV = positive predictive value, NPV = negative predictive value

### Model validation

Internal validation: With internal validation (bootstrap), the AUC was 0.80 (95% CI, 0.77–0.83).

External validation: For the external validation data, the mean ID-BactER score was 3.6 (95% CI, 3.5–3.8, median: 3, interquartile range: 2–5, range of observed scores: 0–10). The relationship between the category of ID-BactER score and the percentage of patients with bacteremia was the same with the validation data as with the derivation data (Fig 2). Specifically, bacteremia was detected in 4.4% (2/45) with a score of 0 or 1; in 10.1% (15/149) with a score of 2 or 3; in 23.9% (32/134) with a score of 4 or 5; in 46.0% (23/50) with a score of 6 or 7; and in 55.6% (5/9) with a score greater than 7. The AUC was 0.74 (95% CI, 0.68–0.80, Fig 3). In contrast, the AUC derived from the model incorporating only temperature was 0.60 (95% CI, 0.54–0.65). The Hosmer-Lemeshow chi-square statistic was 4.17 (p = 0.90). The calibration slope was 1.15 and the intercept was -0.03. Sensitivities, specificities, and likelihood ratios for possible cut-off scores are shown in the lower part of Table 4. With 2 as the cutoff score, the sensitivity was 97%, the specificity was 14%, the positive likelihood ratio was 1.13, the negative likelihood ratio was 0.19, and the percentage of false negatives was 4.4% (2/45).

Complete data from 1709 patients seen from April 2011 through March 2012 were available in derivation and validation data. When using the same cutoff score of 2 in the complete data, the percentage of true positives was 16.7% (285/1709), for false positives it was 67.7% (1157/1709), for false negatives it was 0.4% (6/1709), and for true negatives it was 15.3% (261/1709).

## Discussion

To the best of our knowledge, this study is the first to develop and test a procedure to identify ER patients who have community acquired bacteremia using data available at community hospitals. It uses information on 11 variables, all of which can be obtained easily within a few hours of the time that a patient arrives in the ER of a community hospital. The necessary information comes from the patient’s history, vital signs, physical examination, and results of common blood tests. Unlike other clinical prediction rules for bacteremia, information about bands or procalcitonin is not required. The new procedure results in a score that can range from 0 to 12. With all scores other than 0 and 1 taken as indicators of bacteremia (i.e., test-positive), the sensitivity to true bacteremia in the validation data was 97%.

### Independent predictors compared with existing literature

Most of the predictors used in the new method have previously been found to be associated with community acquired bacteremia. Being older than 65, chills, vomiting, elevated body temperature, hypotension, leukocytosis, thrombocytopenia, and blood urea nitrogen (BUN) were mentioned in a study of ER patients. [17, 18, 22] The strongest predictor in the present analysis, chills, was also the strongest predictor in a review of 35 studies. [12] C-reactive protein (CRP) has been associated with bacteremia in observational studies. [16, 18, 25] However, we identified altered mental status and focal abdominal sign as new predictors to identify community acquired bacteremia.

In contrast, procalcitonin has been used as a predictor of bacteremia [18], but it was not measured at any of the 4 ERs in this study. Measurement of bands [17] was possible at only 1 of the 4 hospitals in this study. In future studies, this biomarker could increase the diagnostic performance of the score.

We compared 3 prediction models previously developed in ER settings (Table 5). [17, 18, 22] Each of the three used data from a single institution. The predictors used by Shapiro [17] were similar to those we used in this study, although Shapiro also used bands. Their resulting AUCs were similar to those in the present study: 0.80 for the derivation data and 0.75 for the validation data. The AUC in the study by Su [18] was slightly higher (0.85), but the internal validation (bootstrap method) resulted in a low AUC (0.66). The number of patients whose data were used in that study was relatively small (n = 558) and there was no external validation. Studying patients with fever, Lee [22] used predictors some of which were the same as those we used in the present study, and the resulting AUC was high: 0.91. However, the number of patients whose data were used in that study was quite small (n = 396) and there was no external validation.

**Table 5:** Three existing models and our model developed in ER settings.

	Shapiro NI, et al. (2008)[17]		Su CP, et al. (2011)[18]		Lee CC, et al. (2012)[22]		This study (2016)	
	predictor	score	predictor	score	predictor	score	predictor	score
**History**	Age > 65 years old	1			Age > 65 years old	1	Age ≥ 65 years old	1
	Chills	1			Rigors	3	Chills	2
	Vomiting	1			Chills	2	Vomiting	1
	Indwelling vascular catheter	2						
	Suspected endocarditis	3						
**Vital signs**	Body temperature 38.3–39.3°C	1	Body temperature ≥ 38.3°C	1	Body temperature > 39.9°C	1	Altered mental status	1
	Body temperature > 39.4°C	3					Body temperature ≥ 38.0°C	1
	Systolic blood pressure < 90 mmHg	1	Heart rate > 120/min	1			Systolic blood pressure < 90 mmHg	1
			Respiratory tract infection	-2				
**Physical sign**							Focal abdominal sign	1
**Laboratory data**	White blood cells > 18,000/μL	1	Lymphocytopenia (< 0.5 × 10^3^/μL)	2			White blood cells ≥ 15,000/μL	1
	Platelets < 150,000/μL	1			Platelets < 100,000/μL	2	Platelets < 150,000/μL	1
	Cre > 2.0 mg/dL	1	AST > 40 IU/L	1	BUN > 20 mg/dL	2	BUN ≥ 20 mg/dL	1
	Bands > 5%	1	CRP > 10 IU/L	1	BUN/Cre > 16	1	CRP ≥ 10 mg/dL	1
			PCT > 0.5 ng/mL	2				
**AUC**	0.80		0.85		0.91		0.80	

Cre = creatinine, AST = aspartate aminotransferase, CRP = C-reactive protein, PCT = procalcitonin, BUN = blood urea nitrogen, AUC = the area under the receiver operating characteristic curve

### Discrimination and calibration of the prediction model

With the ID-BactER score, the AUCs were 0.80 from the derivation data and 0.74 from the validation data. This has been described as “moderate accuracy”. [29] The high p values of the Hosmer-Lemeshow chi-square statistics (0.66 from the derivation data and 0.90 from the validation data) indicate no statistically significant differences between predicted and observed bacteremia. The calibration slope of approximately 1 and intercept of approximately 0 indicate almost-perfect calibration.

One potential problem in the development of any prediction model is overfitting. For this model, the lack of overfitting is evidenced by the calibration slope of 1.15 from the validation data.[31]

### Clinical relevance and implications

For a bacteremia prediction rule to be clinically useful it must yield very few false negatives, that is, it must be very sensitive. In one study, the median sensitivity that 149 physicians required for a bacteremia prediction rule was 95%. [33] When scores ≥ 2 were taken as indicators of bacteremia (i.e., test-positive), the sensitivity of this model was 98% with the derivation data and 97% with the validation data, and the negative likelihood ratios were 0.10 and 0.19 respectively. With that cut-off, false negatives were extremely rare: 1.8% (derivation data) and 4.4% (validation data). This prediction rule meets clinicians’ need for a very sensitive indicator of bacteremia. Therefore we would recommend that clinicians obtain blood-culture results if the ID-BactER score is ≥ 2. If the score is 0 or 1, then there may be no need to draw blood for cultures. Still, with a pre-test probability of 0.1 the post-test probabilities were 0.01 (derivation data) and 0.02 (validation data). [34] Therefore, even with scores of 0 or 1, exceptions might be reasonable (i.e., drawing blood for cultures might be indicated) in some cases. For example, we would not generalize the findings of this study to patients who are suspected to have infectious endocarditis, because very few patients in this study had that diagnosis (0.9% in the derivation data and 0.2% in the validation data).

With a cutoff score of 2 the specificity was 20% for the derivation data and 14% for the validation data. Given a pre-test probability of 0.1, the post-test probability would be 0.12 for the derivation data and 0.11 for the validation data. [34]^34^ The low likelihood ratios do not allow us to “rule in” bacteremia, when using a cutoff score of 2. In addition, that cutoff was associated with 1,157 false-positive results. As noted by Hranjec [35], it is important to minimize the number of unnecessary treatments, and so even if a cutoff score of 2 is used great care must be taken in the prescription of antibiotic drugs. Nonetheless, when a physician strongly suspects that a patient has severe sepsis, then blood for cultures should be drawn as soon as possible and antibiotic treatment should be started early, even before calculating the ID-BactER score.

### Strengths

This study has five strengths. First, this rule was developed and tested with data from randomly selected samples of all patients who underwent blood cultures in the ERs studied, so it is applicable very broadly. It can be used whether the patient originally had only a urinary tract infection, or pneumonia, or cellulitis, etc. Second, all of the data required for the ID-BactER score are available within a few hours of presentation at the ER of a community hospital, even if the patient arrives at night. We note that all of the measurements needed were available at the institution from which the validation data were obtained. Third, for validation we used external data, which is done only rarely despite being very important in this context. [36] Fourth, we used a robust criterion to diagnose bacteremia (i.e., results of 2 or more blood cultures). With that criterion most cases of bacteremia are detected and true bacteremia can be distinguished from contamination. [37] Finally, the outcome was judged by two physicians with more than 10 years of experience in general practice, one of whom was certified as an infectious-disease specialist.

### Limitations

Our study has at least the following eight limitations. The first is the possibility of spectrum bias. [38] The number of patients who in fact had bacteremia could be higher than the number reported here, because in some patients with bacteremia blood cultures might not have been taken. These patients, if they had been included in the study, might have had low ID-BactER scores, and their non-inclusion (because of a lack of blood-culture data) could have caused overestimation of the score’s sensitivity. One challenge for future research is to include all emergency-room patients. The difference in contamination rates between the derivation data and the validation data (35% vs. 25%) could reflect different approaches of clinicians with regard to drawing blood cultures and may limit interpretation of the score’s performance. The second limitation is missing data. [39] The data that were most commonly missing were respiratory rate (24.6%) and LDH (23.7%). For all other candidate predictors fewer than 5% of the data were missing. However, neither respiratory rate nor LDH was an independent predictor of bacteremia in previous studies, and neither was associated with true bacteremia in univariate analysis in this study. So, we believe that missing data did not influence the main result of this study. Third, the study was retrospective, which made it difficult to ensure the quality of the data. As examples, it is possible that not all of the physicians asked about chills, and it is possible that not all of them evaluated each patient’s level of consciousness. Fourth, with the cutoff set at a score of 2, the resulting sensitivity is very high but the specificity is low, which makes the score inappropriate for confirming bacteremia. Future work should focus on building a model that maintains the desired sensitivity and yet is also more specific. Fifth, some physicians may find the score impractical to use when an emergency department is very busy, as the 11 variables could be difficult to remember. Devising a simpler, more easily computed score is another important goal of future work. Sixth, with regard to the sample size we note that, very unfortunately, “there are no generally accepted approaches to estimate the sample size requirements for derivation and validation studies of risk prediction models.” [40] Thus, as described above in the Methods section, our estimate of “about 1500” was based on general considerations for all multivariable models (at 10 cases per predictor variable) and it was also based on the estimate that only about 10% of the records to be reviewed would be from patients who actually had bacteremia (which was a conservative assumption). General rules or widely-applicable guidelines for estimating sample size for development and testing of prediction rules would be welcome. Seventh, the model building did not use a standardized method such as stepwise selection. We considered that clinical variable selection could avoid the potential overfitting, but it is controversial whether clinical variable selection is superior to stepwise selection. Finally, we used data from community hospitals only in Japan, which could reduce the external validity. [41] Further testing with data from other countries is certainly needed.

## Conclusion

Using the ID-BactER score, physicians in the ERs of community hospitals can identify the patients who are most likely to have community acquired bacteremia. The ID-BactER score can be computed from information that is readily available in the ERs of community hospitals. A score that maintains the ID-BactER’s current sensitivity but also has a higher specificity should be developed in future studies. In addition, further validation testing should focus on all emergency-room patients and should use data from different countries.

## Supporting Information

S1 Dataset Dataset of ID-BactER. (XLSX)
